# Supplementary material for: Direct observation of ultrafast exciton localization in an organic semiconductor with soft X-ray transient absorption spectroscopy
Source: Nat Commun. 2022 Jun 14;13:3414. doi: 10.1038/s41467-022-31008-w (PMC9198071; doi:10.1038/s41467-022-31008-w)
Supplement: Supplementary file 1 — Supplementary Information [file 41467_2022_31008_MOESM1_ESM.pdf]

Supplementary Information:

Direct Observation of Ultrafast Exciton Localization in an Organic Semiconductor with Soft X-ray Transient Absorption Spectroscopy

D. Garratt[1*], L. Misiekis[1], D. Wood[1], E. W. Larsen[1], M. Matthews[1], O. Alexander[1], P. Ye[1], S. Jarosch[1], C. Ferchaud[1], C. Strüber[1], A. S. Johnson[1], A. A. Bakulin[2], T. J. Penfold[3], J. P. Marangos[1]

[1]Quantum Optics and Laser Science Group, Blackett Laboratory, Imperial College London, London, SW7 2BW, UK.

[2]Department of Chemistry and Centre for Processable Electronics, Imperial College London, London, W12 0BZ, UK.

[3]Chemistry - School of Natural and Environmental Sciences, Newcastle University, Newcastle upon Tyne, NE1 7RU, UK

*Correspondence to: d.garratt15@imperial.ac.uk

Supplementary Note 1: Measurements at the Sulfur L Edge

Here we present the results from an additional set of measurements performed at the sulfur L edge. Since the absorption cross section at this edge is significantly lower than that at the carbon K edge, these measurements were performed with thicker samples (approximately 200 nm) to optimize the signal to noise ratio at this edge. The Ti filters used in the C K edge measurements to filter the fundamental driving field after the harmonic generation and to block the pump beam before the spectrometer were replaced with Zr in order to increase the flux at around 165 eV. The low transmission of the Zr filters above ~210 eV precludes recording the carbon K edge absorption spectrum simultaneously in these measurements.

At the sulfur $L_{2,3}$ edge there are three pre-edge absorption features which primarily correspond to spin orbit split sulfur 2p $\rightarrow$ σ^*(C-S) transitions with a very weak contribution (<3%) to the highest energy peak from a sulfur 2p $\rightarrow$ π^* transition (1). This can be understood as a consequence of atomic dipole selection rules: In a molecular orbital picture, the π^* orbital is primarily a combination of sulfur $3p_z$ and carbon $2p_z$ orbitals. Since the sulfur 2p $\rightarrow$ 3p transition is dipole forbidden any absorption amplitude at this edge is due to the weak contribution from sulfur 3d orbitals to the π^* orbital. Therefore, this edge is not strongly sensitive to electron density changes at the sulfur site following photoexcitation of the $\pi \rightarrow \pi^*$ transition at 2.25 eV. Instead, we mainly expect any differential absorption signal at this edge to reflect structural/geometrical changes in the polymer in the vicinity of the sulfur atoms.

The raw pumped and unpumped transmission spectra of P3HT at the sulfur L edge are shown in Supplementary Fig. 1a along with the corresponding differential change in absorption (Supplementary Fig. 1b). Here the shaded regions correspond to 1 standard error in the differential absorption. In the pumped spectra we observe a reduction in amplitude and broadening of the absorption peaks, giving rise to three positive and three negative differential absorption features. The time evolution of the differential absorption spectrum is between -200 and +300 fs shown in Supplementary Fig. 1c along the time dependence of the differential absorption signal integrated across each of the positive peaks (Supplementary Fig. 1d). Within the signal to noise ratio of the measurement, there are no significant time dependent changes in the differential absorption signal and the signal persists at negative time delays. This therefore strongly suggests that the signal is due to reversible heating of the samples by the pump pulse. Simulations of the pump induced temperature change presented in Supplementary Fig. 8 indicate that the steady state temperature of the samples is 180°C during the pumped acquisitions. The heating induced broadening therefore dominates the signal at this edge because, as discussed above, it is insensitive to photoexcitation.

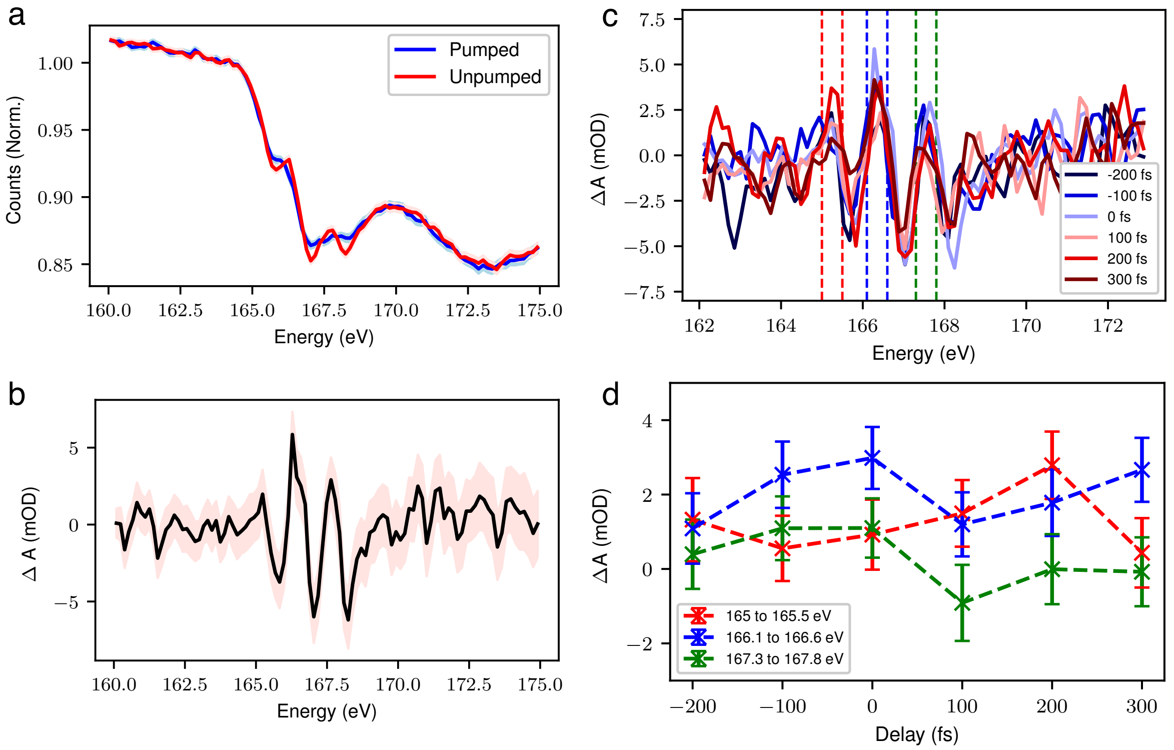

Supplementary Fig. 1. Sulfur $L_{2,3}$ edge measurements. (a) Pumped and unpumped X-ray transmission spectra at the sulfur $L_{2,3}$ edge at zero time delay. The shaded regions indicate ±1 standard error in the measurement. (b) The corresponding differential absorption spectrum at zero time delay. Shaded regions again indicate ±1 standard error. (c) The differential absorption spectrum for time delays between -200 and +300 fs in 100 fs steps. The dashed lines indicate the integration regions used in panel D. (d) The integrated signal across each positive peak in the differential absorption spectrum as a function of time delay. Error bars correspond to ±1 standard error.

Supplementary Note 2: Sample Damage Control Measurements

In addition to being sensitive to thermal heating of the samples by the pump pulse, the sulfur L edge is also sensitive to long term damage of the samples. Supplementary Fig. 2a shows sulfur $L_{2,3}$ edge X-ray absorption spectra recorded before and after exposure to pump beam with a fluence of 1300 ± 60 μJ/cm^2, above the sample damage threshold, for 60 minutes. The spectra are normalized to the magnitude of the $L_{2,3}$ absorption edge. The three absorption peaks broaden and become less distinct indicating a structural change in the polymer. The pump pulse fluence used for these tests was the maximum available in the current setup, but it is reasonable to assume that increased pump intensities will lead to a further broadening and decrease in absorption feature magnitude. This perhaps due to a reduction in geometrical order in the films, however further investigation into the microscopic damage mechanisms is required. Similar effects, although more dramatic, have been observed in the sulfur $L_{2,3}$ edge absorption spectra of bithiophene monolayers deposited on Ag surfaces after exposure to high intensity, white synchrotron radiation, and was attributed to a polymerization reaction which would form disordered layers (*2*). Supplementary Fig. 2b shows the sulfur $L_{2,3}$ edge absorption spectrum recorded before and after the measurements detailed in the main paper. While the noise level in these spectra is higher, a damage induced broadening of all peaks is not observed, indicating that the samples have not undergone significant damage.

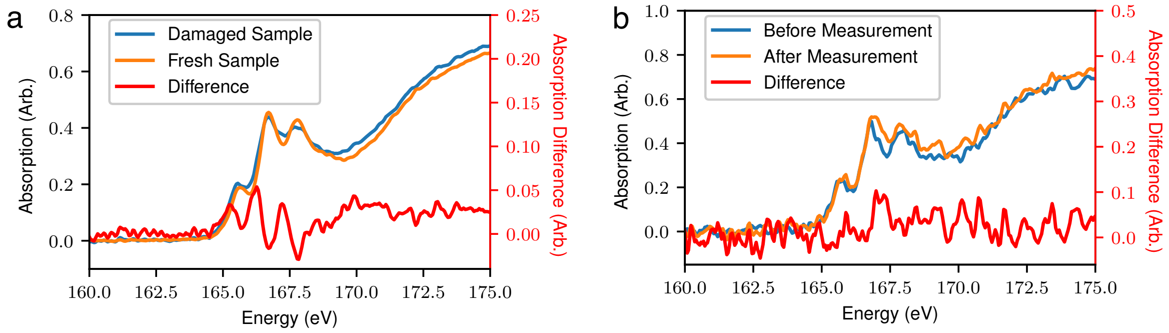

Supplementary Fig. 2. Sulfur $L_{2,3}$ edge sample damage controls (a) The X-ray absorption spectra of P3HT samples at the sulfur $L_{2,3}$ edge before and after exposure to a pump pulse fluence of 1300 ± 60 μJ/cm^2 for 60 minutes. (b) Sulfur $L_{2,3}$ edge absorption spectra recorded before and after the measurements presented in the main paper (Fig. 2).

Supplementary Note 3: Oligomer Chain Length Dependence

The simulations presented in the main paper were performed for isolated and π stacked tetrathiophene oligomers consisting of 4 repeated thiophene units. Supplementary Fig. 3 shows the differential absorption spectrum for isolated thiophene oligomers with chain lengths between 2 and 6 repeated units. Increasing the chain length reduces the energy of the differential absorption feature consistent with an increased conjugation length. The differential absorption spectrum does not change significantly between the tetrathiophene and hexathiophene oligomers in the region of feature A. We therefore choose to use a tetrathiophene oligomer since this allows us to also explore the π stacking dependence of this spectral region at a consistent level of theory without prohibitive computational cost.

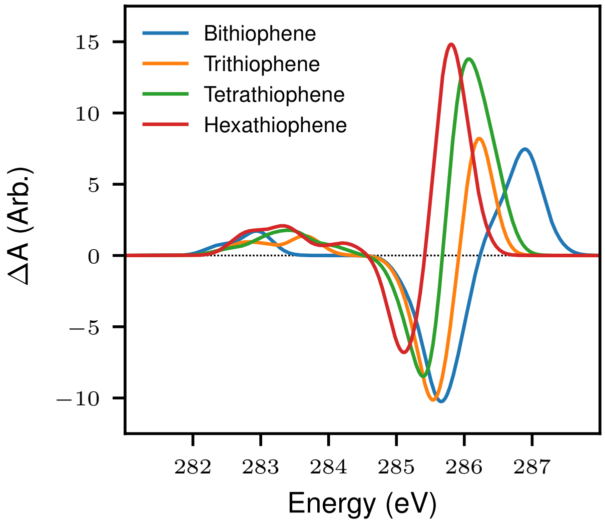

Supplementary Fig. 3 Oligomer chain length dependence. The simulated differential absorption spectrum for an isolated thiophene oligomer chains consisting of 2, 3, 4 and 6 repeat thiophene units.

Supplementary Note 4: Stacking Dependence

In addition to the dimer simulations presented in the main paper we have performed simulations for tetrathiophene oligomer trimer and tetrathiophene oligomer tetramer. The

stacking distance was 0.34 nm. Supplementary Figs. 4a and 4b show the electron density difference between the ground state and the lowest lying triplet state. The corresponding differential X-ray absorption spectra are shown in Supplementary Fig. 4c. Increasing the number of π stacked oligomers increases the extent interchain delocalization leading to a further blue shift and increase in amplitude of the A feature. Note that calculating the X-ray absorption spectrum for the tetrathiophene oligomer trimer and tetrathiophene oligomer tetramer above energies of 285.5 eV is too computationally expensive at present. Therefore the lack of the high energy positive peak in the differential profile of these spectra is not physical.

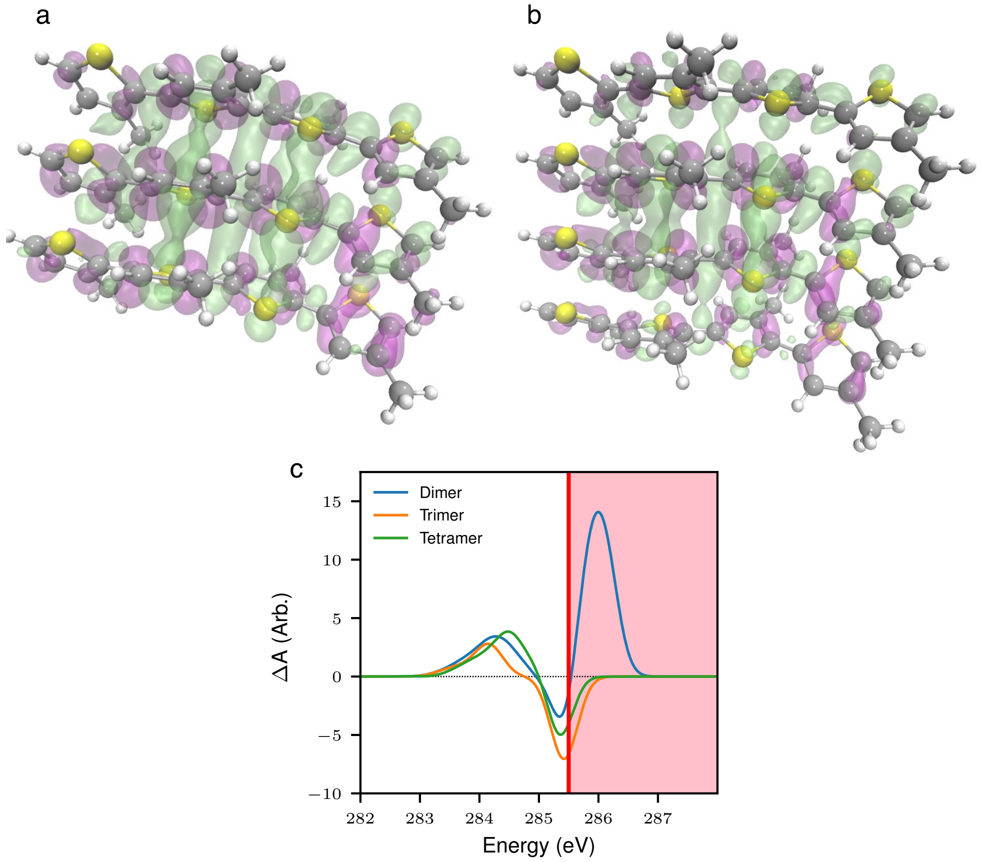

Supplementary Fig. 4 Stacking Dependence. (a, b) Electron density difference plots for three and four π stacked tetrathiophene oligomers (tetrathiophene oligomer trimer and tetramer). (c) Corresponding differential absorption spectra for each system. Above 285.5 eV (red shaded region) the tetrathiophene oligomer trimer and tetramer calculations are not accurate due to the >150 states are required to produce the spectral features in this region.

Supplementary Note 5: The Effect of Nuclear Motion

The effect of nuclear geometry on the simulations is investigated further by comparison of the differential absorption signal in a tetrathiophene oligomer at fixed nuclear geometry and that where the excited state nuclear relaxation has been performed (see Supplementary Fig. 5). The difference between the two transient signals is small and primarily manifests as a small spectral shift in the pre-edge differential absorption, without an increase in amplitude.

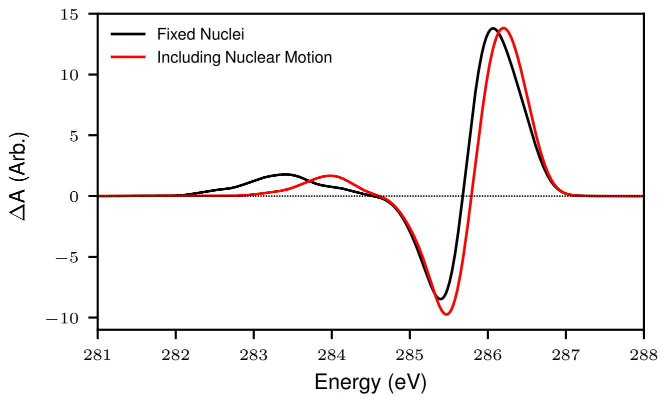

Supplementary Fig. 5. The effect of nuclear motion. Simulated differential absorption spectrum for a tetrathiophene oligomer including the excited state nuclear relaxation (red line) and for fixed nuclei (black line).

Supplementary Tables

Orbital Character of the Monomer and Dimer Excited States

	Energy, Oscillator Strength	Dominant Transition Character
T1	2.32	HOMO-LUMO (0.88)
S1	3.43, f=1.389	HOMO-LUMO (0.99)

Supplementary Table 1. The energy and character of the low lying singlet and triplet excited states of the tetrathiophene oligomer calculated using TDDFT(PBE0).

	Energy (eV), Oscillator strength	Dominant Transition Character
T1	1.88	HOMO-LUMO (0.88)
T2	2.25	HOMO-LUMO+1 (0.6)
T3	2.48	HOMO-LUMO+2 (0.5)
T4	2.73	HOMO-LUMO+3 (0.5)
S1	2.37, f=0.001	HOMO-LUMO (0.99)
S2	2.99, f=0.080	HOMO-LUMO+1 (0.6)
S3	3.13, f=0.0142	HOMO-1-LUMO+1 (0.7)
S4	3.31, f=0.010	HOMO-LUMO+2 (0.99)

Supplementary Table 2. The energy and character of the low lying singlet and triplet excited states of the tetrathiophene oligomer dimer calculated using TDDFT(PBE0).

Supplementary Figures

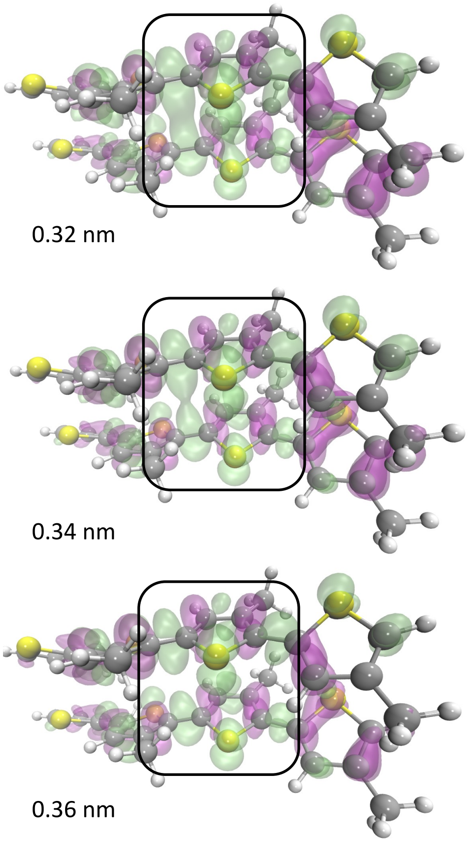

Supplementary Fig. 6 Separation dependent density difference. Density difference of the lowest triplet state for the model tetrathiophene oligomer dimer as a function of the separation, d, between the two oligomers.

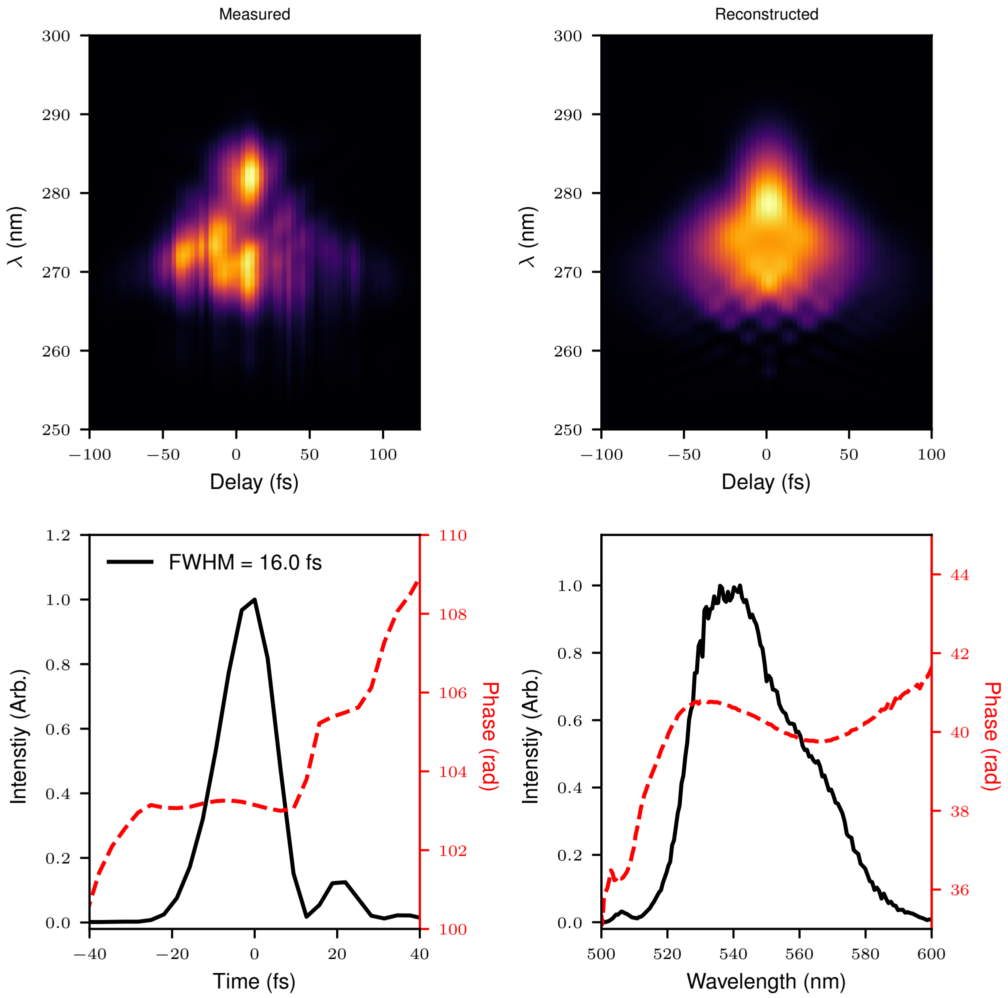

Supplementary Fig. 7 Pump pulse characterization. Measured and reconstructed FROG spectrogram of the visible pump pulse along with the reconstructed temporal and spectral intensity and phase. The measurement gives a pulse duration of 16 fs (FWHM).

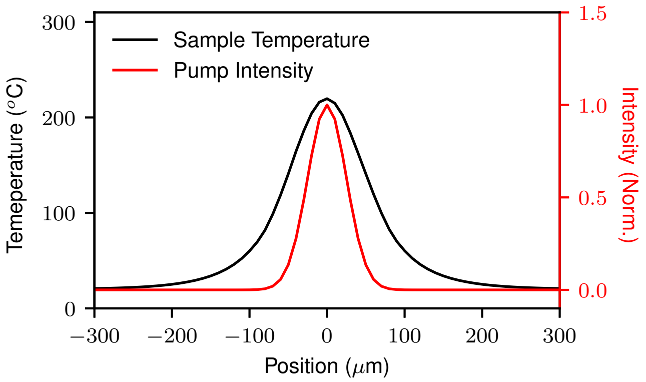

Supplementary Fig. 8. Sample temperature distribution. The radial temperature distribution of the P3HT sample illuminated by an 80 µW CW laser beam, corresponding to 52 µW absorbed power (black line) along with the simulated pump intensity profile (red line). The average temperature across the radius of the probe pulse is 180 °C.

Supplementary References

1. O. D. Baseggio, M. Toffoli, M. Stener, G. Fronzoni, M. de Simone, C. Grazioli, M. Coreno, A. Guarnaccio, A. Santagata, M. D'Auria, S2p core level spectroscopy of short chain oligothiophenes. *The Journal of chemical physics* **147**, 24 (2017).
2. P. Väterlein, M._Schmelzer, J. Taborski, T. Krause, F. Viczian, M. Bäßler, R. Fink, E. Umbach, W. Wurth, Orientation and bonding of thiophene and 2,2′-bithiophene on Ag(111): a combined near edge extended X-ray absorption fine structure and Xα scattered-wave study, *Surface Science*, **452**, 1–3, 20-32 (2000).
